# The Effects of Environment and Physiological Cyclicity on the Immune System of *Viperinae*

**DOI:** 10.1100/2012/574867

**Published:** 2012-04-01

**Authors:** Lorand Kobolkuti, Daniel Cadar, Gabor Czirjak, Mihaela Niculae, Timea Kiss, Carmen Sandru, Marina Spinu

**Affiliations:** ^1^Department of Infectious Diseases, University of Agricultural Sciences and Veterinary Medicine, Manastur street 3-5, 400374 Cluj-Napoca, Romania; ^2^Leibniz Institute for Zoo and Wildlife Research, 10315 Berlin, Germany

## Abstract

One of the important aspects of species' survival is connected with global climate changes, which also conditions the epidemiology of infectious diseases. Poikilotherms are exposed, as other species, to climatic influence, especially due to their physiological peculiarities such as important stages of their life cycle: hibernation, shedding, and active phase. The immune system serves as an accurate indicator of the health status and stress levels in these species. This study aimed to monitor the changes of innate (leukocyte subpopulations and total immune globulins) and adaptive immunity (*in vitro* leukocyte blast transformation) of two viper species, *V. berus berus* and *V. ammodytes ammodytes*, endemic in Europe and spread in different regions of Romania during their three major life cycles, hibernation, shedding, and active phase. The results indicated that seasonal variance and cycle rather than species and regional distribution influence the functionality of the immune system.

## 1. Introduction

The reptilians, “diamonds from ancient worlds” [1] play an important role in the global ecosystem and are considered by the IUCN (The International Union for the Conservation of Nature and Natural Resources) to be biological sensors for pollution with biotic or nonbiotic agents. Since the ′70s, the world of researchers has begun to notice that, globally speaking, the amphibian and reptilian populations experienced a rough decline in numbers, followed by a partial [1] or sometimes total [2] extinction. In Romania, the decline of species of amphibians and reptiles continues [3] despite repeated warnings from the scientific community and the enforcement of conservation laws on endangered species.

An important problem of the ecology is the incomparable way in which environmental factors influence the dynamics of infectious diseases in natural populations of amphibians and reptiles [4]. Currently, it is unanimously agreed that the climate change is influencing the poikilotherm organisms both directly and indirectly [4]. The macroclimate change (global warming) modulates through its direct action the periodicity and the harmony of the immune system [5], whose performance is directly dependant on climate factors. In parallel to direct action, climate change intervenes on the populations of cold-blooded vertebrates indirectly, by changes induced in the ecology of microorganism populations, thus facilitating the emergence of new infectious diseases or reemergence of extinct diseases [2, 6]. The usefulness of the information concerning the detailed positive or negative influence of the macro- and microclimate conditions on the reptiles' immune system, especially in the endangered species of both temperate and tropical climates, is undeniable [1, 7].

In reptilians in general, the functionality of the immune system is supposed to be well adapted to rhythmical climate change [5], with seasonal fluctuations [8–12], and also sensitive to internal and external signals such as hormone levels and photoperiod and temperature, respectively [13].

Decreased immune competence was recorded during the winter and mating seasons, due to the energy intake directed towards survival and reproduction, respectively [5]. Romania has a temperate climate with hot summers and cold winters, as well as intermediate temperature transition seasons, spring and fall, providing a good model for seasonal fluctuation of climatic factors that can influence free ranging reptiles.

Therefore, the study aimed to verify the true versus false character of two hypotheses.

1. In vipers, the functional ability of the immune system fluctuates seasonally, reaching three different protective levels in three major biological cycles: hibernation, shedding, full activity.
2. There are qualitative and quantitative differences in functioning of the immune system of snakes from* Viperidae* related by phylogeny, but distributed in different geoclimatic habitats.

## 2. Materials and Methods

The experiment was carried out on two viper species, naturally inhabiting different regions of Romania, to evaluate the influence of hibernation, shedding, and full activity as well as habitat on both innate and adaptive immunity, by monitoring the dynamics of heterophiles and lymphocytes, total immune globulins, circulating immune complexes, and the *in vitro* blast transformation indices of leukocytes as an anti-infection protective response.

### 2.1. Biological Material

Five common European adders (*Vipera berus subsp. berus*) [3] represented one of the groups, while the second group was composed of five nose-horned vipers (*Vipera ammodytes subsp. ammodytes*). Free ranging animals of the first group are typical for the Carpathian Basin and Eastern Romania while those of the second group are present exclusively in South-Western Romania [14]. The experimental animals were selected from two captive groups of 128 *Vipera berus subsp. berus* and 10 *Vipera ammodytes subsp. ammodytes*, respectively. Both groups of animals came from captive parents, so that their adaptation to captivity was not considered to be an influential factor for the immune system. The animals were kept in terrariums during the entire experiment.

The very primitive thermoregulation system in vipers imposed a very strict temperature control in order to avoid any interference with movements, feeding, sexual cycle, hibernation, and shedding [15]. Thus, the temperature in the terrariums was raised to 20°C with heaters during the day, which were stopped during the night. Heating was also ensured by 40 W light bulbs which also illuminated the terrariums for 14 h/day, from 7 am to 9 pm during the summer and 9 to 10 h/day during spring and fall. The maximal temperature attained in the terrariums was 33-34°C in the “warm pole” and 19-20°C in the “cold pole,” which allowed a behavioral thermoregulation by free movement of the animals.

 The vipers were fed according to their specific needs depending on age, sex, and life cycle [16–19] with laboratory mice and day-old chickens, euthanatized with CO_2_ to avoid venom consumption. They were also offered extra food after shedding, when the interest for feeding increased [20–24].

The number of animals used in procedures was reduced by performing blood samplings on the same animals in dynamics, during the active period, hibernation, and shedding, taking into account the lifetime experience of each individual animal [25]. The procedure of blood sampling has been done at broad intervals, allowing animals to recover after each manoeuvre for a few months.

*Shedding* as a health indicator was monitored according to Mader [15] and was enhanced by the rough ground cardboard surface in the terrariums. The frequency of shedding was monitored according to Biella and Völkl [26].

### 2.2. Hibernation

The starting moment of hibernation depends on the temperature requirements of the viper species. In Romania, *Vipera berus subsp. berus* hibernates later than any other species, sometimes, depending on the outside temperature it can start hibernating in the months of December through March. *Vipera ammodytes ammodytes* is more sensitive to low temperatures; therefore, it hibernates sometimes from September till April. The average temperature during hibernation is 4 to 7°C [27]. In order to ensure hibernating conditions, the animals were kept from November till March at an average of 6 to 7°C, in the same terrariums.

### 2.3. Blood Sampling

Blood was sampled by heart puncture, as recommended by most authors [15, 28], from each *Vipera berus berus* (1 mL) and *Vipera ammodytes ammodytes* (2 mL) during active, shedding, and hibernation periods. Part of the blood was heparinized (50 i.u./mL) and used for leukocyte counts and blast transformation test while the other part was transferred on a procoagulant gel, to obtain the serum. As far as we discerned, vipers recovered fully 1-2 min after blood sampling.

### 2.4. White Blood Cell Count

Blood smears were stained by the use of a panoptic method (Reagens Kft, Budapest), according to instructions for use. Two hundred white cells were counted, and heterophiles and lymphocytes subpopulations were expressed as percentages of the total.

### 2.5. Leukocyte Blast Transformation Test

The leukocyte blast transformation test measures the *in vitro *reactivity of mononuclear cells to sensitizing (*in vivo *encountered) antigens or mitogens [29]. Cell growth was quantified by means of the glucose consumption technique [30]. The blood sample (40 *μ*L) was diluted with four times the amount of RPMI 1640 (Sigma Aldrich ), supplemented with 5% FCS and antibiotics (1000 IU penicillin and 1000 *μ*g streptomycin/mL), for each well of a 96-sterile-wellplate (200 *μ*L per well). Two variants were tested once for each individual animal and sampling period, namely, (1) untreated control culture and (2) phytohaemagglutinin-M (PHA) (Sigma Aldrich) (1 *μ*L per well) treated culture. The culture plates were placed in a 5% CO_2_ atmosphere.

For the hibernation period, the effect of temperature was monitored by incubating the cultures at 4°C and 20°C for 48 h. The blood sampled during the shedding was incubated only at 20°C for the same interval. The samples for the active period were incubated under natural circumstances, that is, in sunshine from 9 to 11 am and 5 to 6 pm; for the rest of the day, the samples were kept in shade at an average of 23°C. Another incubation variant was at 37°C for 48 h.

Glucose concentrations were measured in the initial medium and in all variants at the end of the incubation period, using a standard (100 mg/dL) glucose solution, by means of an ortotoluidine colorimetric test. For this, 12.5 *μ*L of the cultural supernatant were transferred to 0.5 mL of orto-toluidine reagent, boiled for 8 min, cooled suddenly in cold water, and read in a spectrophotometer at 610 nm wavelength (SUMAL PE2, Karl Zeiss, Jena), using the reagent as a blank. The transformation index (IS) was calculated as follows: TI% = [(RG − SG)/RG] × 100, where: IS: blast transformation index, RG: glucose concentration in the RPMI 1640 culture medium, and SG: glucose concentration in the control (M) or phytohaemagglutinin-(PHA-) treated sample after incubation.

### 2.6. Circulating Immune Complex (CIC) Measurements

Measurement of the level of circulating immune complexes (CIC) allows evaluation of the molecular clearance capacity at a particular moment. Part of the collected blood was allowed to clot for 30 min at 37°C and then centrifuged at 1308 g for 10 min. Sera were removed and kept at −20°C until tested. A 4.2% polyethylene glycol (PEG) solution in borate buffer was used as the precipitating agent, while buffer-treated samples served as controls for borate-induced precipitation. The reaction was performed in a 96-well plate to enhance spectrophotometric readings. Volumes of 196.7 *μ*L of borate buffer and PEG solution, respectively, were mixed with 3.3 *μ*L samples of the serum, for each sample, in parallel wells. The samples were allowed to precipitate at room temperature (22-23°C) for 60 min, then read spectrophotometrically at a wavelength of 450 nm in the test plate (*d *= 0.5 cm) (multichannel spectrophotometer SUMAL PE2, Karl Zeiss, Jena, Germany). CIC concentrations, expressed in optical density units (ODU), were calculated by subtracting the value of the control (serum + buffer) from that of the PEG precipitate.

### 2.7. Immunoglobulin Measurements

Total immunoglobulin, known as opsonins, play an important role in the “first line of defense,” that is, innate immunity, against aggressors. At a pH 7.4, the electric charge and colloidal stability of gamma globulins are lower than those of serum albumins. Thus, concentrations as low as 24 mg/L^−1^ of metal salts precipitate the immunoglobulin. A volume of 6.6 *μ*L of serum was mixed with 193.4 *μ*L of a 0.024% barbital buffer zinc sulphate solution and allowed to precipitate for 30 min at room temperature (22-23°C). Optical density (ODU) was then read spectrophotometrically (*λ* = 475 nm, *d *= 0.5 cm).

### 2.8. Statistical Interpretation of the Results

The obtained data were processed using the software Statistica 6.0 by repeated measures ANOVA. The statistical significance of the differences between groups/samplings was interpreted by Tuckey's Honestly Significant Differences test. 

## 3. Results and Discussion

The ontogenetic regulation of the immune system in reptiles conditions maximal protective levels during fully active periods of their lifecycle [5]. The specific immune responses are increased during spring and fall, somewhat decreased during the summer, and sharply decreased during winter, when those reptiles that inhabit temperate regions hibernate and the energy is entirely directed towards ensuring the functioning of cardiovascular energy and survival. Thus, the energy available for other purposes, that is, immune function is symbolic [31]. Therefore, the “hibernation” of the immune system exposes the animal to infections or reinfections with various endogenous or external microbial agents. Nevertheless, this “assumed risk” is vital for poikilotherms in restructuring their major immune organs [5] in order to ensure an appropriate functioning in the subsequent year [32]; indirectly, the seasonal fluctuations of outer conditions direct a harmonically perfectly functioning of defensive systems and subsystems [33].

Physiological peculiarities of the immune system in reptiles, which faithfully reflect the environmental conditions [34], are responsible for the highly stressful nature of these animals; therefore, the remedy of the damage caused by modified environmental conditions should be durable [1]. In reptiles, measurements of white cell subpopulations (micro- and macrophages) as well as dynamics of the blood picture represent a valuable estimate of the protective status of the animal during the major physiological stages (hibernation, shedding, reproduction, etc.) [34, 35].

The interpretation of the blood smear is almost always difficult in reptiles, due to contradictory opinions on white cell types that are present in these animals. Thus, according to Frye [28], reptiles also have neutrophiles not only heterophiles, a view that was not shared by Mader [15] or Murray [36].

**Figure 1:**
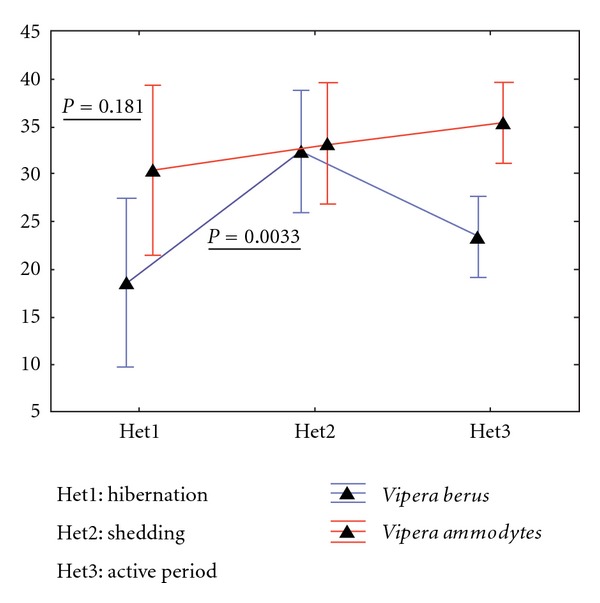
The dynamics of heterophiles cells in *V. berus berus *and* V. ammodytes ammodytes* in various moments of their lifecycle.

Similar to the literature, the investigations indicated that in the studied viper species the heterophiles, “the column of innate immunity” [37], were at a minimal levels during winter and after hibernation; they present a statistically significant increase during shedding, when the cells of adaptive immunity are less functional, as indicated by the decrease of lymphocytes during this period. The differences were statistically significant (*P* = 0.0033) between the sampling periods in the common viper, not in the nose-horned vipers (Figure 1).

**Figure 2:**
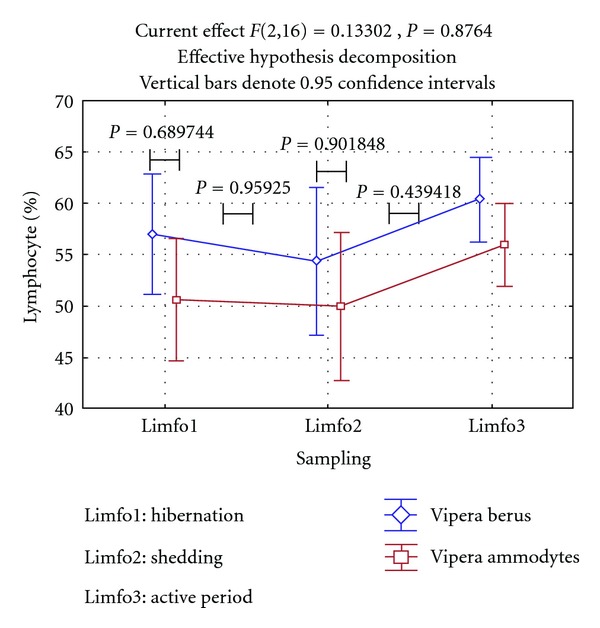
Changes in lymphocyte populations in *V. berus berus *and* V. ammodytes ammodytes *during hibernation, shedding, and active period.

The decreased lymphocyte numbers (Figure 2) appear because part of the mature lymphocytes are removed from general circulation and these cells are entering in the composition of exuvial (shedding) liquid.

During shedding, major part of lymphocytes that remain in the systemic circulation are armed, “reactive,” indicating that shedding represents a major source of stress for vipers. Interestingly, in this study, the percentages of lymphocytes did not decrease dramatically while the animals hibernate, remaining around 50%, a potential cause being the origin of the animals and the relatively similar and constant conditions during all hibernation periods the vipers have been exposed to. 

For the ecologist, high ratios of heterophiles or neutrophiles to lymphocytes (“H : L” or “N : L” ratios) in blood samples reliably indicate high glucocorticoid levels or, simply, stress. This close relationship between stress hormones and N : L or H : L ratios could be highlighted in haematological assessments of stress. Specifically, the changes brought on by stress or glucocorticoid treatment are increases in numbers of neutrophils (neutrophilia) and decreases in lymphocyte numbers (lymphopenia or lymphocytopenia) [38]. In the present study, monitoring the H : L ratios along the experiment indicated that there are differences between the sampling moments and the two groups of animals. Thus, the recorded average values were significantly lower in *V. berus berus* during hibernation (0.347) and the active period (0.380) than in *V. ammodytes ammodytes *during the same sampling moments (0.598 and 0.632, resp.) while the levels for the shedding period were almost similar (0.604 and 0.680, resp.). The values of this indicator supported the strong stressful effect of shedding on both species, but also suggested a higher sensitivity of *V. ammodytes ammodytes *to stressors.

**Figure 3:**
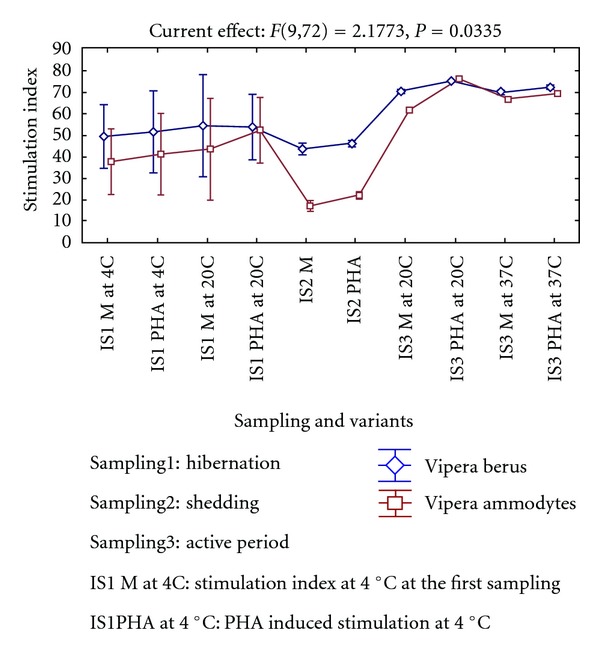
Environmental temperature induced *in vitro* changes in blast transformation indices of leukocyte cultures in common and nose-horned vipers.

With regard to the functionality of cellular adaptive immune system mediated by T lymphocytes, it was noticed that, in the two studied viper species during hibernation at 4°C (Figure 3), the T cells were not able to respond to the mitogen, but the temperature of hibernation is not the major factor that suppressed the functionality of these lymphocytes. Investigations conducted during shedding indicated that the functionality of T lymphocytes in this period was much lower than during hibernation, because a part of lymphocytes disappeared from general circulation and entered into the composition of exuvial liquid. These results were confirmed by the literature indicating that, in temperate climate, in hibernating snakes, the lymphocytes cannot face an infection either due to lack of cell cooperation at low temperatures or the week functionality of this system *in toto* induced by cold [34, 39]. The stimulation indices did not differ significantly during hibernation, when the cultures were incubated at 20°C in neither of the two species or variants. During the shedding period, the blast transformation indices were much lower than during hibernation in both species. The differences between the control versus PHA-stimulated cultures were statistically significant (*P* = 0.002 in *V. berus* and *P* = 0.007 in *V. ammodytes*). The activity of lymphocytes highly increased during the active period, but higher incubation temperatures inhibited rather than stimulated the cell growth (Figure 3).

**Figure 4:**
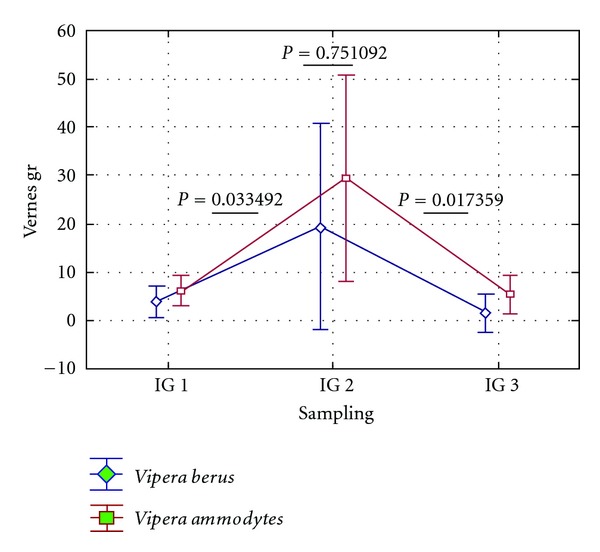
Changes in total gamma globulin concentration during an annual cycle in *V. ammodytes* and *V. berus. *

 Humoral immunity in reptiles, similar to other species, is being mediated by immune globulins or antibodies, as promoters of specific immunity [10]. The lower functionality of cellular adaptive immune system during shedding is counterbalanced by augmentation of humoral immunity, indicated by a significant increase in the concentration of total immune globulins (see Figure 4), as well as circulating immune complexes' levels, which reach maximum levels during shedding. In both species, the levels of CIC were significantly decreased during the active period, as opposed to shedding (*P* = 0.031), supporting the idea that during the latter period cell-mediated immunity was more important for protection.

Immunological investigations showed that there are no relevant qualitative and quantitative differences between the functionality of the immune system in the studied viper species due to geographical distribution, but rather due to their cyclic lifestyle.

## 4. Conclusions

On the basis of the above, the first working hypothesis–the functional ability of the immune system of vipers fluctuates seasonally, reaching three different protective levels in three major biocycles: hibernation, shedding, and activity proved to be true, while the second hypothesis—There are qualitative and quantitative differences in functionality of immune system of *Viperidae* snakes phylogenetically interconnected but distributed in different geoclimatic habitats was false.
